# Successful Functional Outcome in a Dog With Ventricular Tachycardia Treated With Antiarrhythmics, Cardioversion, Cardiopulmonary Resuscitation, and Intra‐Arrest Lipid Emulsion

**DOI:** 10.1111/vec.70089

**Published:** 2026-02-02

**Authors:** Lisa A. Murphy, Sonja S. Tjostheim, Rebecca L. Stepien

**Affiliations:** ^1^ Department of Medical Sciences, School of Veterinary Medicine University of Wisconsin–Madison Madison Wisconsin USA

**Keywords:** arrhythmia, cardiopulmonary resuscitation, defibrillation, electrocardiography, intravenous lipid emulsion

## Abstract

**Objective:**

To describe a case of successful CPR after prolonged cardiopulmonary arrest in a dog treated for refractory ventricular tachycardia (VT) with multiple antiarrhythmic medications and attempted electrical cardioversion, resulting in suspected lidocaine toxicosis necessitating intra‐arrest IV lipid emulsion (ILE) administration.

**Case Summary:**

A 6‐month‐old male intact American Cocker Spaniel was presented for evaluation of VT found on routine physical examination. A dilated cardiomyopathy phenotype was identified on echocardiography. The arrhythmia was refractory to therapy with lidocaine, magnesium sulfate, procainamide, and amiodarone. Subsequently, the dog was anesthetized for electrical cardioversion. The dog then developed ventricular fibrillation (VF), and CPR was performed per the contemporaneous Reassessment Campaign on Veterinary Resuscitation guidelines. Once the duration of VF exceeded 10 min, CPR was adjusted with a longer period of chest compressions before defibrillation. ILE was administered due to a concern for concurrent lidocaine toxicosis and to bind some of the previously administered antiarrhythmic medications, which may have increased the defibrillation threshold (DFT). Return of spontaneous circulation was achieved after 16 min of CPR. After being discharged, the dog was treated with mexiletine and sotalol long term and continued to do well 12 months later despite persistent VT.

**New or Unique Information Provided:**

This report describes a case of CPA secondary to intractable VT refractory to both injectable antiarrhythmic medication and attempted electrical cardioversion. Evidence suggests that antiarrhythmic medications can have positive or negative effects on the DFT, which may affect the success of electrical cardioversion or defibrillation. ILE was administered to bind the lipophilic antiarrhythmic medications due to concern that they were increasing the DFT. In patients with malignant arrhythmias, use of antiarrhythmic medication is often essential; however, clinicians should consider its potential impact on the DFT during subsequent cardioversion or defibrillation.

**Abbreviations**
CPA: cardiopulmonary arrest
CRI: constant rate infusion
DCM: dilated cardiomyopathy
DFT: defibrillation threshold
HR: heart rate
ILE: intravenous lipid emulsion
LQTS: long QT syndrome
PVT: pulseless ventricular tachycardia
RECOVER: Reassessment Campaign on Veterinary Resuscitation
RI: reference interval
ROSC: return of spontaneous circulation
VE: ventricular ectopic
VF: ventricular fibrillation
VT: ventricular tachycardia

## 1 Introduction

Prompt recognition of cardiopulmonary arrest (CPA) and initiation of CPR are required to maximize the likelihood of return of spontaneous circulation (ROSC) [1]. This case report describes the successful resuscitation of a young dog with ventricular tachycardia (VT), prolonged pulseless ventricular tachycardia (PVT), and ventricular fibrillation (VF) using intravenous lipid emulsion (ILE) and cardiac defibrillation. There have been only sparse reports of ROSC with a successful long‐term outcome following prolonged CPA in dogs [2, 3]. The dog in this report received multiple antiarrhythmic medications (lidocaine, magnesium sulfate, procainamide, amiodarone) before electrical cardioversion was attempted. This prompted concern about possible effects of these medications on the defibrillation threshold (DFT), defined as the lowest amount of energy required to successfully defibrillate the heart and restore normal sinus rhythm, both during the attempted electrical cardioversion and subsequent defibrillation during CPR [4].

## 2 Case Summary

**FIGURE 1:**
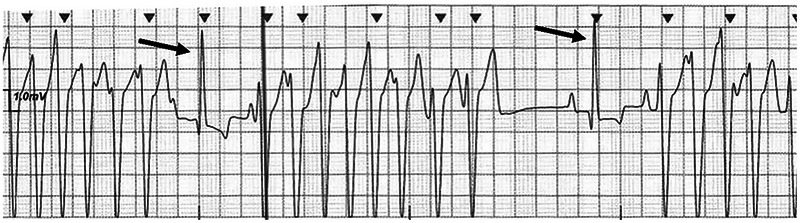
Electrocardiogram of a 6‐month‐old intact male American Cocker Spaniel performed at evaluation before referral. The patient was asymptomatic during the examination. Note repetitive, nonsustained ventricular tachycardia. The average heart rate during ventricular tachycardia was 250/min with the fastest cycle length reaching 300/min. There are two single sinus capture complexes (arrows) between the runs of ventricular tachycardia. Recorded at 25 mm/s, 10 mm/mV.

A 6‐month‐old intact male American Cocker Spaniel weighing 10 kg was presented for evaluation of an arrhythmia found incidentally during pre‐neuter examination. Pulse deficits were noted during the physical examination, and VT was seen on an ECG (Figure 1). Prompt referral to a veterinary cardiologist was recommended, and the dog was presented to the University of Wisconsin's UW Veterinary Care hospital 2 days later. The dog, bred locally and having no travel history, remained asymptomatic during that time. The owner had purchased the dog and two of its littermates (also males) from the same breeder. All three dogs were similar in size and behavior and fed a nontraditional diet containing pulses (peas, lentils, chickpeas, dry beans), potatoes, or sweet potatoes within the top 10 ingredients. No arrhythmia was documented in the other two dogs.

At admission, an ECG was performed, and repetitive, nonsustained, polymorphic VT with evidence of intermittent R‐on‐T phenomenon was documented with a maximum heart rate (HR) of 250/min. All measurements of the sinus complexes were within normal canine reference intervals (RIs), including the QT interval (180 ms; RI: 150–240 ms) [5]. A lidocaine^1^ (2 mg/kg, IV) bolus was administered, and the VT converted into a sinus rhythm for approximately 20–30 s before reverting back to VT. The lidocaine bolus was repeated twice, for a total of three IV boluses, and a lidocaine constant rate infusion (CRI) was initiated at 60 µg/kg/min. During the bolus doses and the initial part of the CRI, the dog displayed short intervals of conversion into a sinus rhythm before VT recurred. A magnesium sulfate^2^ (0.3 mEq/kg, IV) bolus was subsequently administered over 10 min with no discernable effect on the rhythm.

An echocardiogram was performed and revealed moderate left ventricular dilation and mild left ventricular systolic dysfunction consistent with a dilated cardiomyopathy (DCM) phenotype (Table 1) [6]. While receiving the lidocaine CRI, the dog developed sustained VT with a maximum HR of 280/min. A procainamide^3^ bolus (2 mg/kg, IV) was prescribed, but approximately halfway through bolus administration, the rate of VT increased to an HR >300/min. The bolus was discontinued due to concerns that procainamide was having a proarrhythmic effect.

**TABLE 1:** Serial echocardiographic measurements in a young American Cocker Spaniel with DCM phenotype and severe ventricular arrhythmias associated with a diet of pulses, sweet potatoes, and potatoes at baseline, which improved over time with diet change.

	Baseline	2 months after discharge	8 months after discharge	Reference interval [6]
LVIDDn	2.00	1.67	1.56	1.20–1.64
LVIDSn	1.12	1.02	0.83	0.55–0.96
EF%	45%	50%	59.5%	58.9%–63.2%

Abbreviations: DCM, dilated cardiomyopathy; EF%, ejection fraction; LVIDDn, left ventricular internal dimension in diastole normalized to body weight; LVIDSn, left ventricular internal dimension in systole normalized to body weight.

Given that the dog remained in a presumed life‐threatening rhythm despite multiple medications administered, a loading dose of amiodarone^4^ (2 mg/kg, IV) was prescribed with a plan to institute an amiodarone CRI. The amiodarone was also discontinued due to concerns of a proarrhythmic effect, as the dog then developed a persistent R‐on‐T phenomenon after the loading bolus, with an HR >300/min.

By this time, the dog had been receiving the lidocaine CRI for approximately 2 h with a total estimated cumulative dose of 7–9 mg/kg. The dog's mentation had declined, and it was noted to be stuporous. Possible causes for the decline in mentation included lidocaine toxicosis secondary to the multiple antiarrhythmic medications already administered or cerebral hypoperfusion secondary to the severe tachyarrhythmia. The lidocaine infusion was discontinued, and electrical cardioversion was planned.

**FIGURE 2:**
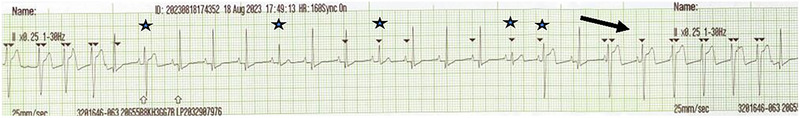
Electrocardiogram of a 6‐month‐old intact male American Cocker Spaniel performed after induction of anesthesia for attempted electrocardioversion. Note the short period of sinus rhythm (heart rate 115/min) followed by resumption of the ventricular rhythm (arrow) with a heart rate of 150/min. Intermittent fusion complexes were also noted (stars). Recorded at 25 mm/s, 10 mm/mV.

**FIGURE 3:**
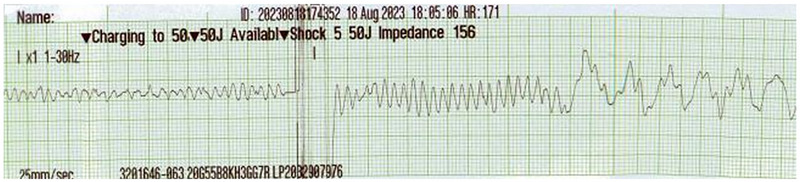
Electrocardiogram of a 6‐month‐old intact male American Cocker Spaniel performed under anesthesia during a pause between CPR chest compression cycles. Note ventricular fibrillation, followed by an external defibrillatory shock of 50 J (5 J/kg) and subsequent ventricular fibrillation. Recorded at 25 mm/s, 10 mm/mV.

Under the guidance of a board‐certified anesthesiologist, the dog was induced approximately 60 min later with midazolam^5^ (0.2 mg/kg, IV) and alfaxalone^6^ (2 mg/kg, IV) and maintained on isoflurane^7^. The thorax was clipped, and pediatric transthoracic pacing pads^8^ were positioned on the dog directly over the palpable cardiac beat. Electrode gel was placed on the pads before being adhered to the dog to minimize electrical resistance, and the pads were secured by circumferentially wrapping the thorax with bandaging tape. Adequate R wave sensing was confirmed on the biphasic defibrillator^9^ to allow for a synchronized shock. After administration of the anesthetic induction agents, the dog was in sinus rhythm for less than 30 s, then reverted to VT (Figure 2). The first synchronized shock (1 J/kg; 10 J) was administered, followed by two escalatory synchronized shocks (20 J, then 30 J), with no conversion to a sinus rhythm. After the third shock was administered, the dog became apneic with no palpable femoral pulse, and VF was noted (Figure 3). CPR was initiated immediately with a protocol of chest compressions between shocks. A fourth external defibrillatory shock of 30 J was administered; this shock was administered as soon as the defibrillator was appropriately charged and was not synchronized due to the presence of VF. Simultaneously, the dog's endotracheal tube was disconnected from the inhalant, and ventilation using an Ambu bag was started per the contemporaneous Reassessment Campaign on Veterinary Resuscitation (RECOVER) guidelines [7]. During this period, the dog's cardiac rhythm alternated between VF and PVT with an HR of 250–300/min. Unlike during the attempted cardioversion, the defibrillatory shocks performed during CPR were not synchronized, but an effort was made to avoid defibrillation on the dog's T wave where possible. ILE^10^ was administered at the onset of CPR due to concerns that lidocaine toxicosis may be present and contributing to CPA, and to attempt to bind any of the previously administered antiarrhythmic medications, which may have increased the DFT. The ILE was administered using a standard protocol of 1.25 mL/kg bolus over 5 min, followed by a further 0.25 mL/kg/min administered over the subsequent 45 min. A protocol of 2 min of chest compressions followed by a defibrillatory shock of 40 J was repeated. Six minutes after starting CPR, the dog developed spontaneous respirations with an increase in end‐tidal CO_2_ from 20 to 50 mm Hg, but no femoral pulse was palpable. The heart rhythm at this time was PVT with an HR of 200/min. Chest compressions were briefly discontinued; however, the dog became apneic again <1 min later, and CPR was restarted with a similar protocol as before. Due to persistent VF, the energy delivered by the defibrillator was increased to a maximum dose of 50 J. After the dog had been in CPA for 10 min, the CPR protocol was adjusted. The dog received a slightly longer period of chest compressions of 3 min, compared with the standard 2 min, before receiving a defibrillatory shock of 50 J. After administration of this shock (delivered 16 min after starting CPR), ROSC was achieved with spontaneous respirations and a palpable femoral pulse. The cardiac rhythm noted upon ROSC consisted of frequent, repetitive, nonsustained runs of VT similar to presentation, as well as occasional runs of accelerated idioventricular rhythm and infrequent sinus complexes; the HR ranged from 140 to 210/min. A fentanyl^11^ bolus (1 µg/kg, IV) was administered to treat for presumed discomfort associated with the high number of defibrillatory shocks administered. This bolus was followed by a fentanyl CRI of 2 µg/kg/h.

Upon recovery from anesthesia, the dog was extubated without incident and transferred to the ICU. The dog continued to have repetitive, nonsustained, polymorphic VT, but noninvasive blood pressures, respirations, and temperature were all normal. Therapy with atenolol^12^ (2 mg/kg, PO, q 12 h) and mexiletine^13^ (7.5 mg/kg, PO, q 8 h) was administered via a nasogastric tube. Atenolol was initially chosen because long QT syndrome (LQTS) was one initial differential diagnosis, and there was a concern for administering sotalol with possible concurrent LQTS.

Over the next 12–24 h, the dog's mentation normalized, and it was discharged 48 h later, receiving sotalol^14^ (2 mg/kg, PO, q 8 h), mexiletine (7.5 mg/kg, PO, q 8 h), and pimobendan^15^ (0.5 mg/kg, PO, q 8 h). The atenolol was discontinued on the day of discharge, and the dog was switched to sotalol as there was no documentation of a prolonged QT interval on any of the multiple ECGs performed in clinic. Transition to a diet that did not contain pulses, potatoes, or sweet potatoes was also recommended. A 48‐h Holter recording was performed 2 weeks later and showed a persistently elevated ventricular ectopic (VE) load of 91%. The dog was evaluated for both the MDR‐1 and KCNQ1 mutations and tested negative for both. The dog also tested negative for toxoplasmosis, neosporosis, and *Bartonella* spp. Whole blood taurine concentration was within normal limits. Twelve months after hospitalization, the dog remained without clinical signs at home with a daily VE load gradually declining based on serial Holter examinations; the most recent total daily VE load (10 months post‐CPR event) was 42%. The dog's diet was changed per previous recommendations, and the DCM phenotype resolved 10 months after diagnosis based on subsequent echocardiograms. Pimobendan was discontinued, but the sotalol and mexiletine doses remained unchanged.

## 3 Discussion

In the current case, multiple antiarrhythmic medications were initially administered in an effort to pharmacologically convert the dog's severe, life‐threatening ventricular tachyarrhythmia to a sinus rhythm. During administration of these drugs, the cardiac rhythm appeared to worsen into intractable rapid VT with concurrent deterioration of the dog's mental status. This response raised concern for proarrhythmia. The proarrhythmic effects of antiarrhythmic medications often manifest as either polymorphic VT or reentrant monomorphic VT by a variety of mechanisms, depending on the drug class [8]. While all antiarrhythmic medications can be proarrhythmic, lidocaine has a lower risk of inducing arrhythmias because it both decreases myocardial heterogeneity and does not prolong the action potential duration, thereby decreasing the risk of reentry and triggered arrhythmias, respectively [9, 10]. In contrast, procainamide slows conduction velocity and increases the action potential duration, both of which can promote development and persistence of VT. In an experimental canine model of myocardial ischemia, the use of procainamide was associated with the development of monomorphic VT in 29% of subjects [11, 12]. In human trials, a proarrhythmic effect that typically manifested as prolongation of the QT interval and rarely progressed to torsades de pointes occurred in 5%–16% of subjects after the administration of procainamide [9, 10, 11, 12]. These effects were suspected to occur secondary to the metabolite *N*‐acetylprocainamide, which has class III activity. Amiodarone, a broad‐spectrum antiarrhythmic medication predominately known for its class III antiarrhythmic effects, blocks delayed rectifier potassium channels (*I*_kr_ and *I*_ks_), which can promote triggered arrhythmias by prolonging the effective refractory period and increasing the action potential duration [13, 14]. Interestingly, amiodarone is associated with a low rate of proarrhythmic effects in people, particularly compared with other class III antiarrhythmic drugs such as sotalol [10]. It has been postulated that this is because amiodarone prolongs the action potential duration to a lesser degree compared with other class III drugs. Ancillary antagonism of calcium channels may also reduce calcium‐dependent afterdepolarizations, which can trigger VT [9, 11, 14]. The incidence of proarrhythmia in dogs treated with amiodarone has not been reported. In the current case, the dog's rhythm appeared unchanged after multiple lidocaine boluses; in contrast, the ventricular rate increased after the procainamide and amiodarone boluses. It is impossible to know if the apparent rhythm changes after the administration of procainamide and amiodarone were secondary to proarrhythmic effects of these medications or represented the natural progression of the malignant ventricular arrhythmia. Regardless, the attending clinicians were prompted to discontinue these medications and attempt electrical cardioversion.

This case involved the use of both electrical cardioversion and defibrillation therapies. The goal of electrical cardioversion is to synchronize the delivery of the electrical current with ventricular depolarization in an attempt to restore sinus rhythm. This is in contrast to defibrillation, in which current delivery cannot be synchronized to the heart rhythm as the individual waveforms are not discernable on the surface ECG. Synchronization during electrical cardioversion is essential in preventing current delivery on the T wave, which can induce VF [15, 16]. The electrical shock should be strong enough to induce a refractory state in approximately 70% of the myocardial mass [16, 17]. With a successful shock, several scenarios can occur. The cardiomyocytes can either enter a prolonged refractory period, or the shock depolarizes excitable cells, which prevents further propagation of the electrical wavefronts [15, 16, 17]. Escalating doses of synchronized current were administered to the dog in this case until VF occurred, necessitating CPR. Although the dog may have entered VF spontaneously, it is also possible that the attempted cardioversion induced VF, despite appropriate synchronization to the R wave. The shock itself can be proarrhythmic if it shortens the refractory period, which restores excitability to the cells. In turn, the cells become the source of a new reentrant wavefront that can deteriorate into VF if there has been sufficient time for the surrounding tissue to have recovered excitability [15, 16].

Although we report a successful outcome after prolonged CPR, it is important to consider any factors that could have affected cardioversion and defibrillation efficacy and necessitate escalation of the current dose. These include inhibition of ion channels (especially sodium and potassium channels) by the multiple, previously administered antiarrhythmic medications, plasma electrolyte concentrations, neurohormonal modulation, gap junction inhibition, and intravascular volume status [18]. It has been shown in human studies that certain antiarrhythmic medications can have positive or negative effects on the DFT, which is the least energy required to successfully defibrillate the heart and restore normal sinus rhythm [18]. A high DFT can become an issue during CPR, as proposed in the current case, and in people with implantable cardiac defibrillators receiving antiarrhythmic medications [19]. The elimination half‐lives of lidocaine, procainamide, and amiodarone in dogs are 0.9, 1.5–4, and 7.5 h, respectively [13, 20, 21]. As such, it is speculated that these drugs were still pharmacodynamically active during the attempted cardioversion and CPR, although the dog's critical illness may have affected normal pharmacokinetics [13]. Sodium channel blockade increases DFT, although this effect can be attenuated in the presence of a concomitant potassium channel blockade [18, 22]. Lidocaine blocks sodium channels without affecting potassium conductance and has been shown to increase the DFT [18]. Drugs that inhibit the delayed potassium rectifier current (*I*_kr_ and *I*_ks_), such as procainamide and amiodarone, can result in prolongation of the action potential duration and have been shown to decrease the DFT [23]. In people, amiodarone and lidocaine both increase the DFT, while procainamide has not been shown to exert a significant effect [23, 24, 25]. Similarly, experimental studies in anesthetized dogs receiving lidocaine showed an increase in DFT of 50%–100% [26]. In contrast, procainamide did not have a significant effect on DFT [21, 27]. Many of these experimental studies used monophasic defibrillators, which have been associated with a higher DFT when compared with biphasic defibrillators [23]. As such, it is possible that the antiarrhythmic medications may exert a more moderate effect than that reported when using a biphasic defibrillator, as was used in this case.

This dog received an ILE during the CPR efforts due to concerns for lidocaine toxicosis, and this may have been a factor in the successful outcome of this case. The ILE may have also cleared the plasma of the previously administered antiarrhythmic medications, theoretically reducing the DFT [28]. The ILE acts as a lipid sink by binding lipophilic drugs, such as lidocaine and amiodarone, while hydrophilic drugs like procainamide are not affected by ILE therapy [13, 29, 30]. The dog's mentation changed from alert to stuporous after receiving multiple boluses of antiarrhythmic medications; this also correlated with worsening of the ventricular arrhythmia. Differential diagnoses for the changes in mentation included drug toxicity, reduced cerebral perfusion due to the persistent rapid VT, or a combination thereof. Lidocaine toxicosis has been associated with central nervous system, cardiovascular, and musculoskeletal signs and can occur with high doses or impaired elimination [31]. In this case, the dog's liver enzymes measured both before and after the CPA event were within normal canine RIs, and it subsequently tested negative for the MDR‐1 mutation, making impaired elimination less likely. Serum lidocaine concentrations were not measured in this dog, but canine studies have reported a toxic dose to be between 6 and 10 mg/kg in dogs, similar to what this dog received [31, 32]. In addition, lipids provide the cardiomyocytes with their primary energy substrate, fatty acids, which can help minimize myocardial ischemia [27]. In veterinary medicine, ILE use during CPA is most commonly associated with local anesthetic toxicosis but could theoretically be beneficial for any lipophilic drug intoxication [28, 29]. In a previous case report, a dog experienced CPA from VT, and CPR with defibrillation was performed for 15 min before the administration of ILE [3], with ROSC achieved 2 min later. In the current case, it was more than 10 min after initiation of ILE before ROSC was achieved. It is therefore difficult to know if the successful resuscitation was due to ILE binding lidocaine and amiodarone, longer duration of or more effective chest compressions, better pharmacologic distribution of the administered resuscitative medications, dissipation of the inhalant anesthetic, or a combination of these factors [13].

Current RECOVER guidelines recommend the immediate initiation of external chest compressions with defibrillation as soon as possible in dogs and cats that experience CPA while an ECG is in place and displays PVT or VF as the initial arrest rhythm [30]. Although older guidelines recommended stacked defibrillation shocks interspersed with chest compressions, newer guidelines advocate for a single defibrillatory shock followed by uninterrupted chest compressions for a full 2‐min cycle, another rhythm check, and defibrillation if indicated [7, 30]. The goal of these newer recommendations is to minimize interruption in chest compressions to optimize myocardial oxygen delivery. VF was recognized immediately in this dog, and therefore, a defibrillatory shock was delivered before the first full 2‐min cycle of chest compressions was completed. After this initial shock, a full 2‐min round of compressions before defibrillation is recommended to optimize myocardial oxygen delivery. This dog initially received CPR per the contemporaneous RECOVER guidelines [7], but we adjusted our CPR protocol once the dog alternated between VF and PVT for 10 min. The decision to perform uninterrupted chest compressions for a longer period of time was made in an attempt to improve myocardial oxygenation because cardiomyocytes are more likely to have normal membrane potentials when they have access to ATP and thus are more likely to respond to electrical defibrillation [33, 34]. However, larger human clinical trials have failed to show that longer uninterrupted chest compression duration is associated with improved survival, and prolonged chest compressions are associated with compressor fatigue, which decays CPR efficacy [35, 36].

After resuscitation, the dog in this case was initially prescribed atenolol and mexiletine for its ventricular arrhythmia. Sotalol administration was not initially pursued due to concerns about possible LQTS. Congenital LQTS was a differential diagnosis for this arrhythmia due to the dog's young age and because the arrhythmia appeared to worsen during with procainamide and amiodarone. LQTS is an electrophysiologic disorder characterized by QT prolongation triggering tachyarrhythmias, typically VT and torsades de pointes. Although multiple genetic mutations have been associated with LQTS in people, only a single mutation in the KCNQ1 gene has been documented in dogs [37]. This genetic mutation may affect normal functioning of the delayed rectifier potassium current, leading to a dispersion of refractoriness, subsequent LQTS, and ventricular arrhythmias [37]. The dog in this case tested negative for the KCNQ1 mutation. Human guidelines for LQTS recommend the use of beta blockers, such as atenolol, while the use of any drug that can prolong the QT interval, such as sotalol, procainamide, and amiodarone, should be avoided [37, 38, 39]. There are no such guidelines in veterinary medicine, although dogs with suspected LQTS have been treated with atenolol [37]. The dog in this case was treated with atenolol while in the hospital after successful resuscitation; however, atenolol was discontinued and sotalol therapy initiated before discharge because a prolonged QT interval was not documented. Thus, LQTS was ultimately considered unlikely. Additionally, the dog continued to exhibit frequent runs of VT with no apparent improvement when receiving the combination of atenolol and mexiletine. Lastly, based on the dog's young age, an inherited ventricular arrhythmia akin to what is described in German Shepherd Dogs was considered a possible etiology [40, 41]. In affected German Shepherd Dogs, a combination of sotalol and mexiletine has proven efficacious in reducing the arrhythmia burden [42]. An inherited ventricular arrhythmia in American Cocker Spaniels has not been previously reported.

The etiology of this dog's ventricular arrhythmia is not known. Differential diagnoses include arrhythmias secondary to an inherited channelopathy such as LQTS (although the test for KCQN1 mutation was negative), infectious disease, or secondary to myocardial fibrosis/ischemia due to a concurrent cardiomyopathy. Primary differential diagnoses for the DCM phenotype included secondary to consumption of a nontraditional diet, secondary to a persistent tachyarrhythmia, or a consequence of chronic myocarditis from infectious or inflammatory disease. Feeding a nontraditional diet has been associated with the development of ventricular arrhythmias and a DCM phenotype, and in some cases, the DCM phenotype is reversible after changing the diet [43]. It is unknown if diet change also reduces the incidence of malignant arrhythmia and sudden death. A scoring system has been created to help identify diets high in pulses, potatoes, and sweet potatoes [43]. Using such a score, this dog's diet was considered to be nontraditional based on the presence of potatoes and sweet potatoes. Primary (genetic) DCM was deemed unlikely due to the improved echocardiographic findings after diet change. Regardless of the exact cause, the rhythm appeared to improve with oral antiarrhythmic therapy, and improvement in myocardial status was seen on echocardiography.

In conclusion, we report the successful resuscitation and survival to hospital discharge with good functional outcome in a dog with VT. The use of multiple antiarrhythmic medications before the development of refractory VT and VF may have influenced the DFT and limited the success of resuscitative efforts until ILE was administered and increased energy was delivered during defibrillatory shocks. The dog in this case remains asymptomatic and continues to be treated with sotalol and mexiletine 12 months after experiencing CPA. The VE load has decreased on subsequent Holter examinations, and the DCM phenotype resolved with a change in diet. In patients with malignant VT, clinicians should be aware of the potential effect some drugs can have on the DFT, particularly if considering synchronous electrical cardioversion or during CPR with defibrillation.

## Ethics Statement

Ethical approval was not required for this manuscript.

## Conflicts of Interest

The authors declare no conflicts of interest.

## Endnotes

1 Lidocaine, Hospira, Inc., Lake Forest, IL.

2 Magnesium sulphate, Fresenius Kabi, Uppsala, Sweden.

3 Procainamide, HR Acquisition Co., LLC Mukilteo, WA.

4 Nexterone, Baxter Healthcare Corporation, Deerfield, IL.

5 Midazolam, Hospira, Inc., Lake Forest, IL.

6 Alfaxalone, Zoetis Inc, Kalamazoo, MI.

7 Isoflurane, Dechra Veterinary Products, KS.

8 Pediatric transthoracic pacing pads, Physio Control, CT.

9 Lifepak biphasic defibrillator, Styker, MI.

10 Intravenous lipid emulsion, Fresenius Kabi, Uppsala, Sweden.

11 Fentanyl, West‐ward, Easttown, NJ.

12 Atenolol, Unichem Pharmaceuticals, East Brunswick, NJ.

13 Mexiletine, Ingenus pharmaceuticals LLC, Orlando, FL.

14 Sotalol, Bayshore pharmaceuticals, Short Hills, NJ.

15 Vetmedin, Boehringer Ingelheim Animal Health USA Inc., Duluth, GA.

## References

[1] D. J. Fletcher and M. Boller , “Fluid Therapy During Cardiopulmonary Resuscitation,” Frontiers in Veterinary Science 7 (2021): 625361, 10.3389/fvets.2020.625361. 33585610 PMC7876065

[2] J. M. Bright and B. D. Wright , “Successful Biphasic Transthoracic Defibrillation of a Dog With Prolonged, Refractory Ventricular Fibrillation,” Journal of Veterinary Emergency and Critical Care 19, no. 3 (2009): 275–279, 10.1111/j.1476-4431.2009.00408. 19691513

[3] A. Mastrocco , A. L. Blutinger , and S. A. Baine , “Use of Injectable Lipid Emulsion and Sodium Bicarbonate to Treat Severe Cardiovascular Collapse Secondary to Lamotrigine Toxicosis in a Dog,” Journal of the American Veterinary Medical Association 258, no. 5 (2021): 510–514, 10.2460/javma.258.5.510. 33620240

[4] S. K. Mainigi and D. J. Callans , “How to Manage the Patient With a High Defibrillation Threshold,” Heart Rhythm 3, no. 4 (2006): 492–495, 10.1016/j.hrthm.2005.12.023. 16567304

[5] R. Santilli , S. Moïse , R. Pariaut , et al., Electrocardiography of the Dog and Cat. 2nd Edition: Diagnosis of Arrhythmias (Edra, 2018).

[6] L. C. Visser , M. M. Ciccozzi , D. J. Sintov , et al., “Echocardiographic Quantitation of Left Heart Size and Function in 122 Healthy Dogs: A Prospective Study Proposing Reference Intervals and Assessing Repeatability,” Journal of Veterinary Internal Medicine 33, no. 5 (2019): 1909–1920, 10.1111/jvim.15562. 31313382 PMC6766555

[7] E. A. Rozanski , J. E. Rush , G. J. Buckley , D. J. Fletcher , M. Boller , and RECOVER Advanced Life Support Domain Worksheet Authors ., “RECOVER Evidence and Knowledge Gap Analysis on Veterinary CPR. Part 4: Advanced Life Support: RECOVER Advanced Life Support,” Journal of Veterinary Emergency and Critical Care 22, no. Suppl. 1 (2012): S44–S64, 10.1111/j.1476-4431.2012.00755. 22676286

[8] J. E. Tisdale , M. K. Chung , K. B. Campbell , et al., “Drug‐Induced Arrhythmias: A Scientific Statement From the American Heart Association,” Circulation 142, no. 15 (2020): e214–e233, 10.1161/cir.00000000000009059. 32929996

[9] L. N. Horowitz , A. M. Greenspan , A. P. Rae , et al., “Proarrhythmic Responses During Electrophysiologic Testing,” American Journal of Cardiology 59, no. 11 (1987): E45–E48, 10.1016/0002-9149(87)90201-3. 3578040

[10] A. E. Buxton and M. E. Josephson , “Role of Electrophysiologic Studies in Identifying Arrhythmogenic Properties of Antiarrhythmic Drugs,” Circulation 73, no. 2 Pt 2 (1986): II67–II72. 3943175

[11] S. H. Hohnloser , “Amiodarone‐Associated Proarrhythmic Effects: A Review With Special Reference to Torsade De Pointes Tachycardia,” Annals of Internal Medicine 121, no. 7 (1994): 529–535, 10.7326/0003-4819-121-7-199410010-00009. 8067651

[12] J. S. Steinberg , D. I. Sahar , M. Rosenbaum , et al., “Proarrhythmic Effects of Procainamide and Tocainide in a Canine Infarction Model,” Journal of Cardiovascular Pharmacology 19, no. 1 (1992): 52–59, 10.1097/00005344-199201000-00008. 1375688

[13] M. G. Papich , Saunders Handbook of Veterinary Drugs: Small and Large Animal, 4th ed. (Elsevier, 2016).

[14] B. N. Singh , “Choice and Chance in Drug Therapy of Cardiac Arrhythmias: Technique Versus Drug‐Specific Responses in Evaluation of Efficacy,” American Journal of Cardiology 72, no. 16 (1993): F114–F124, 10.1016/0002-9149(93)90974-h. 8237824

[15] R. Santilli , R. Pariaut , and M. Perego , Cardiac Arrhythmias in Dogs and Cats (Edizioni LSWR, 2024).

[16] B. Lown , “Defibrillation and Cardioversion,” Cardiovascular Research 55, no. 2 (2002): 220–224, 10.1016/s0008-6363(02)00416-9. 12123757

[17] I. Efimov and C. M. Ripplinger , “Virtual Electrode Hypothesis of Defibrillation,” Heart Rhythm 3, no. 9 (2006): 1100–1102, 10.1016/j.hrthm.2006.03.005. 16945810

[18] A. L. Dopp , J. M. Miller , and J. E. Tisdale , “Effect of Drugs on Defibrillation Capacity,” Drugs 68, no. 5 (2008): 607–630, 10.2165/00003495-200868050-00004. 18370441

[19] E. R. Uyguanco , A. Berger , A. S. Budzikowski , et al., “Management of High Defibrillation Threshold,” Expert Review of Cardiovascular Therapy 6, no. 9 (2008): 1237–1248, 10.1586/14779072.6.9.1237. 18939911

[20] R. Latini , G. Tognoni , and R. E. Kates , “Clinical Pharmacokinetics of Amiodarone,” Clinical Pharmacokinetics 9, no. 2 (1984): 136–156, 10.2165/00003088-198409020-00002. 6370540

[21] M. G. Papich , L. E. Davis , and C. A. Davis , “Procainamide in the Dog: Antiarrhythmic Plasma Concentrations After Intravenous Administration,” Journal of Veterinary Pharmacology and Therapeutics 9, no. 4 (1986): 359–369, 10.1111/j.1365-2885.1986.tb00056.x. 3806778

[22] D. L. Ware , J. B. Atkinson , M. J. Brooks , et al., “Ventricular Defibrillation in Canines With Chronic Infarction, and Effects of Lidocaine and Procainamide,” Pacing and Clinical Electrophysiology 16, no. 2 (1993): 337–346, 10.1111/j.1540-8159.1993.tb01585. 7680463

[23] A. A. Mehdirad , C. A. Carnes , and S. D. Nelson , “The Influence of Specific and Nonspecific Potassium Current Blockade on the Defibrillation Energy Requirement of Biphasic Shock,” Pacing and Clinical Electrophysiology 22, no. 1 Pt 2 (1999): 147–151, 10.1111/j.1540-8159.1999.tb00322. 9990620

[24] W. Jung , M. Manz , L. Pizzulli , et al., “Effects of Chronic Amiodarone Therapy on Defibrillation Threshold,” American Journal of Cardiology 70, no. 11 (1992): 1023–1027, 10.1016/0002-9149(92)90354-2. 1414899

[25] P. Leong‐Sit , L. J. Gula , P. Diamantouros , et al., “Effect of Defibrillation Testing on Management During Implantable Cardioverter‐Defibrillator Implantation,” American Heart Journal 152, no. 6 (2006): 1104–1108, 10.1016/j.ahj.2006.06.02526. 17161062

[26] S. L. Topham , Y. M. Cha , B. B. Peters , et al., “Effects of Lidocaine on Relation Between Defibrillation Threshold and Upper Limit of Vulnerability in Open‐Chest Dogs,” Circulation 85, no. 3 (1992): 1146–1151, 10.1161/01.cir.85.3.1146. 1537112

[27] G. M. Deeb , R. L. Hardesty , B. P. Griffith , et al., “The Effects of Cardiovascular Drugs on the Defibrillation Threshold and the Pathological Effects on the Heart Using an Automatic Implantable Defibrillator,” Annals of Thoracic Surgery 35, no. 4 (1983): 361–366, 10.1016/s0003-4975(10)61585-8. 6838263

[28] S. H. Ok , J. M. Hong , S. H. Lee , et al., “Lipid Emulsion for Treating Local Anesthetic Systemic Toxicity,” International Journal of Medical Sciences 15, no. 7 (2018): 713–722, 10.7150/ijms.22643. 29910676 PMC6001420

[29] S. García‐Ramos , I. Fernandez , and M. Zaballos , “Lipid Emulsions in the Treatment of Intoxications by Local Anesthesics and Other Drugs. Review of Mechanisms of Action and Recommendations for Use,” Revista Española de Anestesiología y Reanimación (English Edition) 69, no. 7 (2022): 421–432, 10.1016/j.redare.2021.03.018. 35871141

[30] J. Wolf , G. J. Buckley , E. A. Rozanski , et al., “2024 RECOVER Guidelines: Advanced Life Support. Evidence and Knowledge Gap Analysis With Treatment Recommendations for Small Animal CPR,” Journal of Veterinary Emergency and Critical Care 34, no. S1 (2024): 44–75, 10.1111/vec.13389. 38924633

[31] V. Vieitez , I. Á. Gómez de Segura , M. Martin‐Cuervo , et al., “Successful Use of Lipid Emulsion to Resuscitate a Foal After Intravenous Lidocaine Induced Cardiovascular Collapse,” Equine Veterinary Journal 49, no. 6 (2017): 767–769, 10.1111/evj.12699. 28502090

[32] N. Lemo , D. Vnuk , B. Radisic , et al., “Determination of the Toxic Dose of Lidocaine in Dogs and Its Corresponding Serum Concentration,” The Veterinary Record 160, no. 11 (2007): 374–375, 10.1136/vr.160.11.374. 17369479

[33] H. J. Choi , T. Nguyen , K. S. Park , et al., “Effect of Cardiopulmonary Resuscitation on Restoration of Myocardial ATP in Prolonged Ventricular Fibrillation,” Resuscitation 84, no. 1 (2013): 108–113, 10.1016/j.resuscitation.2012.06.006. 22727945

[34] M. L. Weisfeldt and L. B. Becker , “Resuscitation After Cardiac Arrest: A 3‐Phase Time‐Sensitive Model,” Journal of the American Medical Association 288, no. 23 (2002): 3035–3038, 10.1001/jama.288.23.3035. 12479769

[35] M. Reyes‐Martínez and V. J. Herrería‐Bustillo , “Evaluation of Compressor Fatigue at 150 Compressions per Minute During Cardiopulmonary Resuscitation Using a Large Dog Manikin,” Journal of Veterinary Emergency and Critical Care 33, no. 5 (2023): 495–500, 10.1111/vec.13331. 37578021

[36] J. W. Heidenreich , R. A. Berg , T. A. Higdon , et al., “Rescuer Fatigue: Standard Versus Continuous Chest‐Compression Cardiopulmonary Resuscitation,” Academic Emergency Medicine 13, no. 10 (2006): 1020–1026, 10.1197/j.aem.2006.06.049. 17015418

[37] W. A. Ware , Y. Reina‐Doreste , and J. A. Stern , “Sudden Death Associated With QT Interval Prolongation and KCNQ1 Gene Mutation in a Family of English Springer Spaniels,” Journal of Veterinary Internal Medicine 29, no. 2 (2015): 561–568, 10.1111/jvim.12550. 25779927 PMC4895492

[38] M. T. Bennett , L. J. Gula , G. J. Klein , et al., “Effect of Beta‐Blockers on QT Dynamics in the Long QT Syndrome: Measuring the Benefit,” Europace 16, no. 12 (2014): 1847–1851, 10.1093/europace/euu086. 24833771

[39] I. A. Khan , “Long QT Syndrome: Diagnosis and Management,” American Heart Journal 143, no. 1 (2002): 7–14, 10.1067/mhj.2002.120295. 11773906

[40] N. Sydney Moïse , R. F. Gilmour , and M. L. Riccio , “Diagnosis of Inherited Ventricular Tachycardia in German Shepherd Dogs,” Journal of the American Veterinary Medical Association 210, no. 3 (1997): 403–410, 10.2460/javma.1997.210.03.403. 9057927

[41] E. Sosunov , “Abnormal Cardiac Repolarization and Impulse Initiation in German Shepherd Dogs With Inherited Ventricular Arrhythmias and Sudden Death,” Cardiovascular Research 42, no. 1 (1999): 65–79, 10.1016/s0008-6363(98)00333-2. 10434997

[42] A. R. M. Gelzer , M. S. Kraus , M. Rishniw , et al., “Combination Therapy With Mexiletine and Sotalol Suppresses Inherited Ventricular Arrhythmias in German Shepherd Dogs Better Than Mexiletine or Sotalol Monotherapy: A Randomized Cross‐Over Study,” Journal of Veterinary Cardiology 12, no. 2 (2010): 93–106, 10.1016/j.jvc.2010.06.001. 20663731

[43] L. Freeman , J. Rush , D. Adin , et al., “Prospective Study of Dilated Cardiomyopathy in Dogs Eating Nontraditional or Traditional Diets and in Dogs With Subclinical Cardiac Abnormalities,” Journal of Veterinary Internal Medicine 36, no. 2 (2022): 451–463, 10.1111/jvim.16397. 35297103 PMC8965249

